# Predicting propofol requirements in advanced gastrointestinal endoscopy: a validated model incorporating age and comorbidity index

**DOI:** 10.3389/fmed.2026.1860431

**Published:** 2026-07-09

**Authors:** Yueh-Juh Lin, Pei-Lin Lin

**Affiliations:** 1 Department of Cardiology, En Chu Kong Hospital, New Taipei City, Taiwan; 2 Department of Anesthesiology, National Taiwan University Hospital, Taipei, Taiwan

**Keywords:** advanced gastrointestinal endoscopy, Charlson Comorbidity Index, prediction model, procedural sedation, propofol

## Abstract

**Background:**

Propofol requirements during advanced gastrointestinal endoscopy vary substantially among patients with multimorbidity, and the influence of comorbidity burden remains incompletely characterized. This study aimed to evaluate the association between Charlson Comorbidity Index (CCI), age-adjusted CCI (ACCI), and propofol requirement, and to develop a model for individualized sedation.

**Methods:**

This retrospective cohort study included 1,555 adult patients undergoing advanced gastrointestinal endoscopy (primarily endoscopic retrograde cholangiopancreatography and endoscopic ultrasonography) under bispectral index (BIS)-guided target-controlled infusion (TCI) propofol sedation at a tertiary referral center between 2017 and 2023. The primary outcome was the lean body mass (LBM)-adjusted propofol infusion rate. Patients were classified into higher- and lower-propofol requirement groups for logistic regression analyses, and sensitivity analyses were performed using the continuous outcome variable. Model discrimination was evaluated using the area under the receiver operating characteristic curve (AUC) with internal split-sample validation.

**Results:**

Older age, male sex, longer procedure duration, and higher CCI were independently associated with a lower probability of higher propofol requirement in multivariable analysis. Sensitivity analyses using the continuous LBM-adjusted propofol infusion rate yielded consistent findings. The model demonstrated good discrimination in both the training cohort (AUC 0.831, 95% CI 0.808–0.853) and validation cohort (AUC 0.841, 95% CI 0.780–0.882).

**Conclusions:**

Older age, male sex, and higher comorbidity burden were independently associated with lower relative propofol requirements during advanced gastrointestinal endoscopy under BIS-guided TCI sedation. The proposed model demonstrated acceptable discrimination; however, external validation is required before clinical application.

## Introduction

With the rapid aging of the global population and continuous advances in therapeutic endoscopic techniques, the number of elderly patients with multiple comorbidities undergoing advanced gastrointestinal endoscopy has increased substantially. Procedures such as endoscopic retrograde cholangiopancreatography (ERCP) and endoscopic ultrasonography (EUS) are often technically demanding and prolonged, requiring deeper and more stable sedation than conventional diagnostic endoscopy. Consequently, propofol-based deep sedation has become a preferred sedation strategy for advanced GI endoscopy because of its rapid onset, short recovery time, and favorable procedural conditions (1–3).

Despite these advantages, propofol administration in medically complex patients remains challenging because excessive sedation may increase the risk of respiratory depression and hemodynamic instability. A large national study reported that cardiopulmonary unplanned events during GI endoscopy were significantly associated with advanced systemic disease and comorbidities (4). These findings suggest that comorbidity burden may influence not only procedural risk, but also individualized sedative requirements.

Previous pharmacokinetic and pharmacodynamic studies have shown that aging and physiological impairment may alter propofol sensitivity and dosing requirements (5, 6). The Charlson Comorbidity Index (CCI) and age-adjusted Charlson Comorbidity Index (ACCI) are widely validated tools for quantifying systemic comorbidity burden and aging-related vulnerability (7–9). However, whether these indices are associated with propofol requirement during advanced GI endoscopy has not been systematically investigated.

Therefore, this study aimed to evaluate the association between comorbidity burden, as quantified by the CCI and ACCI, and propofol requirement during advanced GI endoscopy performed using a target-controlled infusion (TCI) and bispectral index (BIS)-guided sedation strategy. In addition, we sought to identify major clinical covariates associated with propofol requirement and to develop explanatory models for individualized sedation management.

## Methods

### Study population and setting

**Figure 1:**
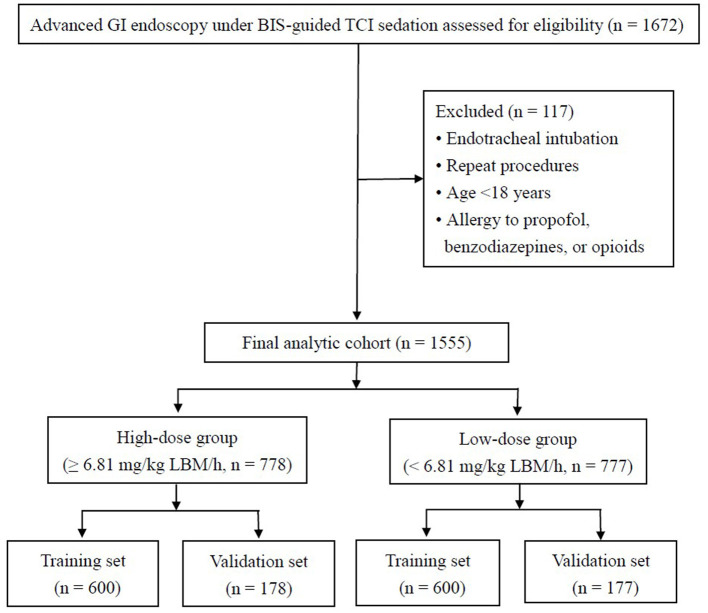
Flowchart of study selection and classification. Patients were screened for eligibility (*n* = 1,672) and excluded according to predefined criteria. The final cohort (*n* = 1,555) was categorized into high- and low-dose groups (cutoff: 6.81 mg/kg LBM/h) and split into training and validation sets. LBM, lean body mass; TCI, target-controlled infusion.

This retrospective cohort study was conducted at National Taiwan University Hospital, a tertiary academic referral center. Adult patients undergoing advanced gastrointestinal endoscopic procedures, primarily ERCP and EUS, under a standardized propofol-based deep sedation protocol between July 2017 and March 2023 were retrospectively identified from the institutional sedation database. The study population was restricted to procedures managed by a single anesthesiologist using a routine BIS-guided target-controlled infusion (TCI) protocol. All procedures were performed by experienced endoscopists at a high-volume tertiary referral center (Figure 1). Only the first oral advanced GI endoscopic procedure for each patient was included in the analysis to ensure independent observations. Patients younger than 18 years, those requiring general anesthesia with endotracheal intubation, and those undergoing repeat procedures were excluded. The final analytic cohort consisted of 1,555 patients.

The study protocol was approved by the Institutional Review Board of National Taiwan University Hospital (No. 202305045RINA).

### Sedation protocol and TCI implementation

All procedures were performed using a previously described propofol-based sedation strategy with a TCI system (Injectomat AGILIA, Fresenius Vial, France) (10). Patients received premedication with intravenous midazolam (2 mg) and alfentanil (0.4 mg) before propofol administration.

Propofol infusion was primarily guided using the Schnider pharmacokinetic model because of its favorable effect-site targeting characteristics. In patients whose BMI exceeded the built-in limits of the TCI device, the Marsh model was alternatively applied to facilitate stable drug delivery.

The initial target plasma concentration (Cp) was set at 1.0 μg/ml and subsequently titrated in increments of 0.2 μg/ml according to clinical sedation depth and BIS monitoring, with a target BIS range of 60–80 corresponding to moderate-to-deep sedation (10). Hemodynamic and respiratory parameters were continuously monitored throughout the procedure and recorded at 5-min intervals.

Rescue interventions for peri-procedural adverse events were performed as clinically indicated. Hypotension was managed with intravenous fluids and norepinephrine when necessary. Intra-procedural hypertension was managed with supplemental opioids and/or antihypertensive agents as clinically indicated. Hypoxemia was managed with airway maneuvers and supplemental oxygen.

### Data collection and outcome measures

Demographic data, procedural characteristics, and comorbid conditions were extracted from electronic medical records. Comorbidity burden was quantified using the CCI and ACCI.

The primary outcome was the lean body mass (LBM)-adjusted mean propofol infusion rate (mg/kg LBM/h), calculated by dividing the total administered propofol dose (induction plus maintenance) by the product of sedation duration and estimated LBM. LBM was estimated using a validated anthropometric formula based on body weight and body mass index (BMI). LBM adjustment was adopted based on pharmacokinetic evidence suggesting that LBM better reflects propofol distribution and clearance than total body weight (5, 6).

For secondary analyses, patients were stratified according to propofol requirements into high- and low-dose groups using the upper tertile cutoff of the LBM-adjusted propofol infusion rate. The high-dose group was defined as patients requiring ≥6.81 mg/kg LBM/h, whereas the low-dose group included those requiring < 6.81 mg/kg LBM/h. This categorization was used to facilitate clinical interpretation and exploratory discrimination analysis.

### Statistical analysis and model development

Continuous variables were assessed for normality using the Kolmogorov–Smirnov test and compared using Student's *t*-test. Categorical variables were analyzed using the chi-square test or Fisher's exact test.

To explore factors associated with high propofol requirement, univariate logistic regression analyses were performed. Variables with clinical relevance and/or a univariate association (*P* < 0.05), including age, sex, Charlson Comorbidity Index (CCI), procedure type, and procedure duration, were considered for multivariable analysis.

A multivariable logistic regression model was constructed to identify independent factors associated with high propofol requirement. Model discrimination was evaluated using the area under the receiver operating characteristic curve (AUC-ROC).

Internal validation was performed using a random split-sample approach, with 77.2% of the cohort assigned to the training set (*n* = 1,200) and 22.8% to the validation set (*n* = 355).

Sensitivity analyses treating propofol requirement as a continuous outcome (mg/kg LBM/h) were performed using linear regression to assess the robustness of the findings.

All statistical analyses were performed using SAS version 9.4 software (SAS Institute, Cary, North Carolina). The study followed the TRIPOD reporting guidelines for prediction model development.

## Results

A total of 1,555 patients undergoing advanced gastrointestinal endoscopic procedures were included in the final analysis. Procedures consisted primarily of ERCP and EUS, as well as other complex interventions, including endoscopic submucosal dissection, enteroscopy-based therapeutic procedures, and duodenal stent placement for gastric outlet obstruction.

For descriptive comparison, the training cohort (*n* = 1,200) was stratified into high- and low-dose groups according to the median LBM-adjusted mean propofol infusion rate. Baseline characteristics differed between groups. Compared with the high-dose group, patients in the low-dose group were older, had longer procedure duration, higher ASA class, greater comorbidity burden, and a higher prevalence of cardiovascular and metabolic comorbidities, malignancy, and hypertension. Table 1 summarizes baseline characteristics of the training cohort.

**Table 1:** Baseline clinicodemographic characteristics and comorbidity profiles.

Characteristics	High-dose group (≥6.81 mg/kg LBM/h) (*n* = 600)	Low-dose group (< 6.81 mg/kg LBM/h) (*n* = 600)	*P* value
Sex, Female/male, *n*	400/200	199/401	< 0.001^*^
Age, mean (SD), y	56.64 (14.27)	68.67 (12.28)	< 0.001^*^
Procedure type			0.001^*^
EUS	392 (65.33%)	327 (54.50%)	
ERCP	167 (27.83%)	223 (37.17%)	
EUS+ERCP	29 (4.83%)	30 (5.00%)	
Others	12 (2.00%)	20 (3.33%)	
ASA			< 0.001^*^
1	32 (5.33%)	8 (1.33%)	
2	450 (75.0%)	324 (54.0%)	
3	118 (19.67%)	253 (42.17%)	
4	0 (0.00%)	15 (2.50%)	
Procedure duration, mean (SD), min	29.42 (18.62)	38.38 (22.23)	< 0.001^*^
CCI, mean (SD)	1.54 (2.02)	2.58 (2.37)	< 0.001^*^
ACCI, mean (SD)	2.91 (2.56)	4.98 (2.74)	< 0.001^*^
Myocardial infarction, *n* (%)	24 (4.00%)	80 (13.33%)	< 0.001^*^
Congestive heart failure, *n* (%)	15 (2.50%)	43 (7.17%)	< 0.001^*^
Peripheral vascular disease, *n* (%)	0 (0.00%)	9 (1.50%)	0.004^*^
Cerebrovascular disease, *n* (%)	11 (1.83%)	26 (4.33%)	0.012^*^
Dementia, *n* (%)	3 (0.50%)	14 (2.33%)	0.007^*^
Chronic obstructive pulmonary disease, *n* (%)	28 (4.67%)	27 (4.50%)	0.890
Connective tissue disease, *n* (%)	21 (3.50%)	11 (1.83%)	0.073
Peptic ulcer disease, *n* (%)	79 (13.17%)	121 (20.17%)	0.001^*^
Mild liver disease, *n* (%)	95 (15.83%)	95 (15.83%)	1.000
DM without end-organ damage, *n* (%)	91 (15.17%)	189 (31.50%)	< 0.001^*^
DM with end-organ damage, *n* (%)	0 (0.00%)	4 (0.67%)	0.124
Hemiplegia, *n* (%)	2 (0.33%)	9 (1.50%)	0.034^*^
Moderate to severe chronic renal disease, *n* (%)	15 (2.50%)	22 (3.67%)	0.242
Malignant solid tumor, *n* (%)	95 (15.83%)	154 (25.67%)	< 0.001^*^
Lymphoma, *n* (%)	5 (0.83%)	10 (1.67%)	0.194
Leukemia, *n* (%)	1 (0.17%)	2 (0.33%)	1.000
Moderate to severe liver disease, *n* (%)	9 (1.50%)	21 (3.50%)	0.027^*^
Metastatic solid tumor, *n* (%)	49 (8.17%)	78 (13.00%)	0.007^*^
Hypertension, *n* (%)	189 (31.50%)	304 (50.67%)	< 0.001^*^

LBM, lean body mass; EUS, endoscopic ultrasonography; ERCP, endoscopic retrograde cholangiopancreatography; ASA, American Society of Anesthesiologists; CCI, Charlson comorbidity index; ACCI, age-adjusted Charlson comorbidity index. ^*^P < 0.05 considered statistically significant.

The majority of patients (*n* = 1,548) were managed using the Schnider pharmacokinetic model, while a small proportion (*n* = 7; high-dose group: 6 patients; low-dose group: 1 patients) received the Marsh model due to device limitations in patients with extreme BMI. Norepinephrine use was infrequent and comparable between groups (high-dose group: 9 patients; low-dose group: 15 patients). No severe sedation-related adverse events requiring pharmacological or mechanical intervention, including atropine administration or manual bag-mask ventilation, were observed during the study period. No missing data were present in the dataset; therefore, no imputation was required.

### Multivariable model development

Univariate logistic regression analyses identified age, sex, procedure duration, and CCI as factors associated with propofol requirement (Table 2). These variables were subsequently entered into the multivariable logistic regression model.

**Table 2:** Univariable and multivariable logistic regression analyses of factors associated with relative propofol requirements (mg/kg LBM/h).

Variable	Univariable analysis		Multivariable analysis (model 1)		Final multivariable model	
	OR (95% CI)	*P* value	aOR (95% CI)	*P* value	aOR (95% CI)	*P* value
Sex						
Female	1 (ref)		1 (ref)		1 (ref)	
Male	0.248 (0.195–0.316)	< 0.001	0.166 (0.123–0.223)	< 0.001	0.169 (0.126–0.226)	< 0.001
Age	0.934 (0.925–0.944)	< 0.001	0.924 (0.912–0.936)	< 0.001	0.924 (0.913–0.935)	< 0.001
Procedure type						
EUS	1 (ref)		1 (ref)			
ERCP	0.625 (0.487–0.801)	< 0.001	1.255 (0.911–1.730)	0.165	—	—
EUS+ERCP	0.806 (0.474–1.371)	0.427	1.126 (0.564–2.248)	0.737	—	—
Others	0.501 (0.241–1.039)	0.063	1.036 (0.412–2.606)	0.941	—	—
Procedure duration (min)	0.978 (0.972–0.984)	< 0.001	0.981 (0.974–0.988)	< 0.001	0.982 (0.975–0.989)	< 0.001
ASA						
1	1 (ref)		1 (ref)			
2	0.347 (0.158–0.763)	0.009	1.176 (0.484–2.855)	0.720	—	—
3	0.117 (0.052–0.261)	< 0.001	1.073 (0.410–2.807)	0.887	—	—
4	Not estimable		Not estimable		Not estimable	
CCI	0.803 (0.759–0.849)	< 0.001	0.943 (0.878–1.014)	0.114	0.931 (0.873–0.992)	0.028

Model 1 included variables selected based on univariable analyses (*P* < 0.05) together with prespecified clinically relevant covariates (age, sex, procedure type, procedure duration, ASA classification, and CCI). The final multivariable model was derived using backward elimination with a removal criterion of *P* > 0.05. The age-adjusted Charlson Comorbidity Index was excluded because of collinearity with the Charlson Comorbidity Index (Pearson's r = 0.898). ASA class IV (*n* = 20, 1.3%) was not estimable in the logistic regression models because of sparse data and quasi-complete separation. Ref, reference; EUS, endoscopic ultrasonography; ERCP, endoscopic retrograde cholangiopancreatography; ASA, American Society of Anesthesiologists; CCI, Charlson comorbidity index.

In multivariable analysis, older age, male sex, longer procedure duration, and higher CCI were independently associated with a lower probability of higher propofol requirement after adjustment for covariates (Table 2). A strong correlation was observed between ACCI and CCI (Pearson's r = 0.898); therefore, ACCI was excluded from the final model due to multicollinearity.

The final logistic regression model for estimating the probability (P) of higher propofol requirement was defined as:

logit (*P*) = 6.6560 – 1.7792 × Sex (male = 1, female = 0) – 0.0792 × Age (per year) – 0.0184 × Procedure duration (per minute) – 0.0718 × Charlson Comorbidity Index score.

Where *P* represents the probability of higher propofol requirement.

The probability was calculated using the inverse logit function:


$$\begin{array}{l} P=\frac{1}{1+\;e^{-logit\left(P\right)}} \end{array}$$

### Model discrimination

**Figure 2:**
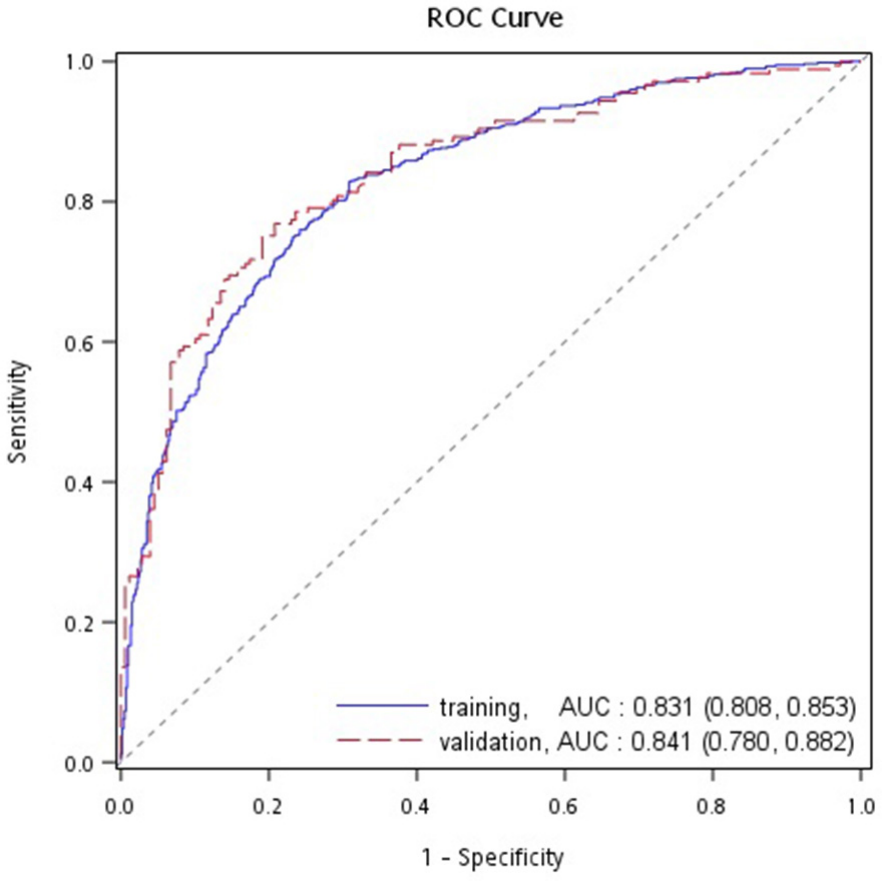
Receiver operating characteristic (ROC) curves for the prediction of propofol requirements in the training and validation datasets. The area under the curve (AUC) was used to assess model discrimination.

The multivariable model demonstrated moderate discriminatory performance for identifying patients with lower propofol requirement. In the training cohort, the area under the receiver operating characteristic curve (AUC-ROC) was 0.831 (95% CI: 0.808–0.853). In the validation cohort, the AUC-ROC was 0.841 (95% CI, 0.780–0.882) (Figure 2).

Using the optimal cutoff determined by the Youden index, the model demonstrated a sensitivity of 0.791%, specificity of 0.747%, positive predictive value of 0.757%, and negative predictive value of 0.782% in the validation cohort (Table 3).

**Table 3:** Summary of model performance metrics.

Dataset	*N*	AUC (95% CI)	Sensitivity	Specificity	PPV	NPV
Training	1,200	0.831 (0.808, 0.853)	0.828	0.692	0.729	0.801
Validation	355	0.841 (0.800, 0.882)	0.791	0.747	0.757	0.782

Performance metrics in the training and validation datasets include area under the receiver operating characteristic curve (AUC), sensitivity, specificity, positive predictive value (PPV), and negative predictive value (NPV). Metrics were calculated using an optimal probability threshold of 0.417, determined by the Youden index (0.52) derived from the training set ROC curve.

### Sensitivity analyses

Sensitivity analyses using the continuous outcome variable (mg/kg LBM/h) yielded results generally consistent with those of the primary logistic regression model. Older age, male sex, and higher comorbidity burden remained independently associated with lower relative propofol requirement in multivariable linear regression analyses (Supplementary Table S1).

Additional analyses adjusting for procedure duration and procedure type to account for potential procedural confounding demonstrated similar effect estimates, with the association between greater comorbidity burden and lower propofol requirement remaining statistically and directionally consistent.

## Discussion

This study identified independent associations of older age, male sex, and higher comorbidity burden with lower relative propofol requirements during advanced gastrointestinal endoscopy under standardized BIS-guided TCI sedation. These findings were consistent across internal validation and sensitivity analyses using the continuous outcome measure.

The association between higher comorbidity burden and lower relative propofol requirement is biologically plausible. Chronic systemic disease may alter sedative drug response through changes in organ perfusion, protein binding, pharmacokinetics, and metabolic reserve (5). Compared with the ASA physical status classification, which is subject to interobserver variability (11), the CCI provides a cumulative and quantitative measure of comorbidity burden that may better reflect overall physiological reserve in elderly patients with multimorbidity (7, 9). Higher CCI scores may also capture frailty-related vulnerability, which has been associated with adverse postoperative outcomes in prospective cohort studies (12). Such reductions in physiological reserve could increase sensitivity to propofol and contribute to lower sedative requirements.

Because adiposity may confound total body weight–based normalization of anesthetic dosing, the primary outcome was expressed as the LBM-adjusted propofol infusion rate. This approach was intended to reduce adiposity-related variability in propofol distribution and pharmacokinetics (5, 13).

Male sex was independently associated with lower relative propofol requirements. Although the underlying mechanisms were not specifically evaluated, prior pharmacokinetic studies have demonstrated sex-related differences in propofol distribution, clearance, and body composition that may influence anesthetic requirements (5). Older age was associated with lower propofol requirements, consistent with previous studies demonstrating reduced propofol clearance and increased pharmacodynamic sensitivity in elderly patients (14).

The present findings may help identify patient characteristics associated with lower relative propofol requirements during advanced gastrointestinal endoscopy. However, given the observational design and absence of external validation, the model should be interpreted primarily as an explanatory rather than clinically applicable predictive model. Nevertheless, these findings are consistent with broader efforts in perioperative medicine to develop individualized approaches to anesthetic risk stratification based on preprocedural patient characteristics (15).

Strengths of this study include the relatively large cohort size, the use of a previously established BIS-guided TCI sedation protocol, and incorporation of clinically relevant comorbidity assessment using the CCI within a real-world advanced gastrointestinal endoscopy population. The use of an LBM-adjusted outcome improved pharmacokinetic interpretability by accounting for interindividual differences in body composition. In addition, sedation was administered under a standardized protocol with minimized variability in anesthetic delivery, thereby enhancing internal validity. Finally, inclusion of multiple advanced endoscopic procedures, including ERCP, EUS, and other complex interventions, supports the clinical generalizability of the findings within tertiary care settings.

Several limitations should be acknowledged. First, this was a retrospective single-center study, which may limit generalizability. Although procedure duration and procedure type were included in the regression analyses, residual confounding from unmeasured procedural and clinician-dependent factors may remain. In addition, anesthesiologists may have adjusted propofol titration according to patient characteristics, and continuous BIS data were not available for retrospective analysis. Second, all patients received standardized pre-procedural midazolam and alfentanil, and their independent contribution to propofol requirements could not be isolated. Third, although the Schnider model was used in the vast majority of patients, alternative pharmacokinetic models such as the Eleveld model (5) were not evaluated. Finally, external validation was not available, and validation in independent cohorts is required before clinical implementation.

In conclusion, older age, male sex, and higher comorbidity burden were independently associated with lower relative propofol requirements during advanced gastrointestinal endoscopy under BIS-guided target-controlled infusion sedation. The proposed model demonstrated acceptable discrimination in the study cohort; however, external validation is required before clinical application.

## Data Availability

The original contributions presented in the study are included in the article/Supplementary material, further inquiries can be directed to the corresponding author.
