# Supplementary material for: Predicting propofol requirements in advanced gastrointestinal endoscopy: a validated model incorporating age and comorbidity index
Source: Front Med (Lausanne). 2026 Jul 9;13:1860431. doi: 10.3389/fmed.2026.1860431 (PMC13391333; doi:10.3389/fmed.2026.1860431)
Supplement: Supplementary file 1 [file Supplementary_file_1.docx]

| Supplementary Table S1. Univariable and multivariable linear regression analyses of factors associated with relative propofol requirements (mg/kg LBM/h). | | | | | | | | |
| --- | --- | --- | --- | --- | --- | --- | --- | --- |
| Variable | Univariable analysis | |  | Multivariable analysis (Model 1) | |  | Final multivariable model | |
|  | Univariable β (95% CI) | P value |  | Multivariable β (95% CI) | P value |  | Multivariable β (95% CI) | P value |
| Sex |  |  |  |  |  |  |  |  |
| Female | 1 (Ref) |  |  | 1 (Ref) |  |  | 1 (Ref) |  |
| Male | -2.072 (-2.393, -1.750) | <0.001 |  | -1.988 (-2.263, -1.713) | <0.001 |  | -1.996 (-2.270, -1.722) | <0.001 |
| Age | -0.097 (-0.107, -0.087) | <0.001 |  | -0.089 (-0.100, -0.078) | <0.001 |  | -0.092 (-0.102, -0.083) | <0.001 |
| Procedure type |  |  |  |  |  |  |  |  |
| EUS | 1 (Ref) |  |  | 1 (Ref) |  |  |  |  |
| ERCP | -0.879 (-1.248, -0.510) | <0.001 |  | 0.140 (-0.174, 0.455) | 0.382 |  | — | — |
| EUS+ERCP | -0.521 (-1.316, 0.275) | 0.199 |  | -0.034 (-0.692, 0.625) | 0.921 |  | — | — |
| Others | -1.102 (-2.163, -0.042) | 0.042 |  | -0.055 (-0.920, 0.811) | 0.902 |  | — | — |
| Procedure duration (min) | -0.033 (-0.041, -0.025) | <0.001 |  | -0.020 (-0.026, -0.013) | <0.001 |  | -0.020 (-0.027, -0.013) | <0.001 |
| ASA |  |  |  |  |  |  |  |  |
| 1 | 1 (Ref) |  |  | 1 (Ref) |  |  |  |  |
| 2 | -1.864 (-2.769, -0.959) | <0.001 |  | -0.310 (-1.088, 0.467) | 0.434 |  | — | — |
| 3 | -3.664 (-4.593, -2.735) | <0.001 |  | -0.534 (-1.402, 0.333) | 0.227 |  | — | — |
| 4 | -6.057 (-7.747, -4.367) | <0.001 |  | -2.451 (-3.948, -0.954) | 0.001 |  | — | — |
| CCI | -0.340 (-0.413, -0.267) | <0.001 |  | -0.069 (-0.139, 0.001) | 0.054 |  | -0.096 (-0.160, -0.032) | <0.001 |

Model 1 included variables selected based on univariable analyses (P < 0.05) together with prespecified clinically relevant covariates (age, sex, procedure type, procedure duration, ASA classification, and CCI). The final multivariable model was derived using backward elimination with a removal criterion of P > 0.05. The age-adjusted Charlson Comorbidity Index was excluded because of collinearity with the Charlson Comorbidity Index (Pearson’s r = 0.898).

Ref, reference; EUS, endoscopic ultrasonography; ERCP, endoscopic retrograde cholangiopancreatography; ASA, American Society of Anesthesiologists; CCI, Charlson comorbidity index.
